# Updated rapid risk assessment from ECDC on coronavirus disease (COVID-19) pandemic in the EU/EEA and the UK: resurgence of cases

**DOI:** 10.2807/1560-7917.ES.2020.25.32.2008131

**Published:** 2020-08-13

**Authors:** Eurosurveillance editorial team

**Affiliations:** 1 European Centre for Disease Prevention and Control (ECDC), Stockholm, Sweden

The European Centre for Disease Prevention and Control (ECDC) provides regularly updated information on coronavirus disease (COVID-19) relevant to Europe on a dedicated webpage. Besides general information including infographics, daily case counts, and maps with disease distribution, examples of latest updates comprise: COVID-19 clusters and outbreaks in occupational settings in the EU/EEA and the UK, Objectives for COVID-19 testing in school settings, and COVID-19 in children and the role of school settings in COVID-19 transmission. ECDC also publishes regular risk assessments and the Box below contains the summary from the eleventh update published on 10 August 2020.

Box **Summary of the ECDC rapid risk assessment from 10 August 2020**

The COVID-19 pandemic continues to pose a major public health threat to EU/EEA countries and the UK and to countries worldwide. As cases increased, peaking in early April 2020 in the EU/EEA, many countries implemented a range of response measures which led to a reduction in incidence. As countries regained control of transmission and alleviated the burden on healthcare, many measures were relaxed or removed to allow for a more viable way of life with the virus in circulation. Subsequently, a recent increase in COVID-19 cases has been reported in many EU/EEA countries. While many countries are now testing mild and asymptomatic cases, which has resulted in increased case reports, there is a true resurgence in cases in several countries as a result of physical distancing measures being relaxed.

Further increases in the incidence of COVID-19, and associated hospitalisations and deaths, can be mitigated if sufficient control measures are reinstalled or reinforced in a timely manner. Countries that are now observing an increase in cases, after having lifted their control measures following a temporary improvement in the epidemiological situation, should consider re-instating selected measures through a phased, step-wise and sustainable approach. Assessment of risk at local level is important, taking into consideration the epidemiological situation, local services and lessons learned regarding the impact of previous measures.

Member States implementing comprehensive testing are better able to rapidly detect an increase in cases and identify groups at high risk of disease. Alongside a tailored local testing strategy, the speed of contact tracing is important to reduce transmission, and efforts should be made to shorten the time needed for each step in the testing, notification, and contact tracing process.

Given that there are now dedicated COVID-19 surveillance systems, extensive public health measures in place, and ongoing testing and contact tracing of the population, countries should be better prepared to prevent and control any resurgence in cases.

In general, response strategies should be guided by continuous monitoring and assessment of the epidemiological situation. They should be based on sustainable public health measures to protect vulnerable groups and decrease transmission in the community and should include extensive testing and contact tracing, followed by isolation and treatment of identified cases and quarantining of contacts. In addition to the preparedness and response strategies implemented by national authorities, adapted human behaviour is the key to tackling this pandemic. As the COVID-19 pandemic continues, it is natural for people to become fatigued and reduce compliance with public health measures. Risk communication efforts should be tailored to changes in the local situation and continuous messaging is needed to remind the population that the SARS-CoV-2 virus will remain in circulation within the community and that they should take everyday measures to reduce potential exposure, such as practising cough and respiratory etiquette, physical distancing and hand hygiene, wearing face masks, reducing the number of contacts and staying home when ill.

**What is new in this update?**

- Updated epidemiological situation and response measures implemented in the EU/EEA countries and the UK.
- Updated testing strategies, contact tracing, and general and targeted measures to minimise the risk of COVID-19 resurgence.
- Various risk profiles, based on the changes countries are observing in their reported cases, hospitalisations, testing methodologies, and test positivity rates in response to the relaxing or removing of measures.

**What are the risks being assessed in this update? **

In this update, we analyse the risk of further escalation of COVID-19 in the countries that have reported a recent increase in COVID-19 cases and the risk of further escalation of COVID-19 across all EU/EEA countries and the UK.

In countries where there is a strong indication of increasing transmission, locally or nationally, as demonstrated by a recent increase in cases and an increase in hospitalisations, the risk of further escalation of COVID-19 is **high**. For those countries, the risk is **very high** if they do not implement or reinforce multiple measures, including physical distancing and contact tracing, if they have sufficient testing capacity.

In countries where there is evidence that is suggestive of increasing transmission, as demonstrated by a recent increase in cases and no increase in hospitalisations but where there has been an increase in test positivity rates (if they have sufficient testing capacity and intensity of testing has remained stable), the risk of further escalation is **high**. For those countries, the risk is **very high** if they do not implement or reinforce multiple measures, including physical distancing and contact tracing.

The risk of further escalation of COVID-19 is **moderate to high** for countries reporting a recent increase in cases but no increase in hospitalisations or test positivity rates (if they have sufficient testing capacity and intensity of testing has remained stable). Countries that have multiple measures in place should conduct local assessments to better understand the local drivers of the increase in cases and to determine measures to be added or strengthened.

Overall, the risk of further escalation of COVID-19 across all EU/EEA countries and the UK (if they have sufficient contact tracing and testing capacity), is **moderate** for countries that continue to implement and enforce multiple measures including physical distancing and **very high** for countries that do not implement or enforce such measures.

Source: European Centre for Disease Prevention and Control (ECDC). Rapid risk assessment: Coronavirus disease 2019 (COVID-19) in the EU/EEA and the UK – eleventh update: resurgence of cases. Stockholm: ECDC; 10 Aug 2020. Available from: https://www.ecdc.europa.eu/en/publications-data/rapid-risk-assessment-coronavirus-disease-2019-covid-19-eueea-and-uk-eleventh
COVID: coronavirus disease; ECDC: European Centre for Disease Prevention and Control; EU/EEA: European Union/European Economic Area; SARS-CoV: severe acute respiratory syndrome coronavirus; UK: United Kingdom.

